# Effects of low back pain on balance performance in elderly people: a systematic review and meta-analysis

**DOI:** 10.1186/s11556-021-00263-z

**Published:** 2021-06-05

**Authors:** Le Ge, Chuhuai Wang, Haohan Zhou, Qiuhua Yu, Xin Li

**Affiliations:** 1 Department of Rehabilitation Medicine, The First Affiliated Hospital, Sun Yat-sen University, Zhongshan Road, Guangzhou, 510080 Guangdong Province China; 2 Pathophysiology Department of Basic Medical Collage, Jilin University, Changchun, China

**Keywords:** Low back pain, Balance performance, Elderly people

## Abstract

**Background:**

Research suggests that individuals with low back pain (LBP) may have poorer motor control compared to their healthy counterparts. However, the sample population of almost 90% of related articles are young and middle-aged people. There is still a lack of a systematic review about the balance performance of elderly people with low back pain. This study aimed to conduct a systematic review and meta-analysis to understand the effects of LBP on balance performance in elderly people.

**Methods:**

This systematic review and meta-analysis included a comprehensive search of PubMed, Embase, and Cochrane Library databases for full-text articles published before January 2020. We included the articles that 1) investigated the elderly people with LBP; 2) assessed balance performance with any quantifiable clinical assessment or measurement tool and during static or dynamic activity; 3) were original research. Two independent reviewers screened the relevant articles, and disagreements were resolved by a third reviewer.

**Results:**

Thirteen case-control studies comparing balance performance parameters between LBP and healthy subjects were included. The experimental group (LBP group) was associated with significantly larger area of centre of pressure movement (*P* < 0.001), higher velocity of centre of pressure sway in the anteroposterior and mediolateral directions (*P* = 0.01 and *P* = 0.02, respectively), longer path length in the anteroposterior direction (*P* < 0.001), slower walking speed (*P* = 0.05), and longer timed up and go test time (*P* = 0.004) than the control group.

**Conclusion:**

The results showed that balance performance was impaired in elderly people with LBP. We should pay more attention to the balance control of elderly people with LBP.

## Background

It was reported that the world’s population aged ≥60 years will triple by 2050 [1]. Rapidly growing aging populations have increased the prevalence of diseases such as musculoskeletal pain. The reported prevalence of muscular and skeletal pain is 65–85% in elderly people [2, 3], 36–70% of which had LBP [3, 4]. Low back pain was the most common health problem among older adults, results in pain and disability [5]. Moreover, elderly people with LBP are often underreported and inadequately provided with treatment [6]. Untreated or undertreated older individuals with LBP may experience sleep disturbances, limitations to their social and recreational activities, psychological distress, decreased cognition, rapid deterioration of functional ability, and falls subsequently causing great burdens on family and society [7–9].

Balance, which is fundamental to activities of daily living, is impaired in the patients with LBP [10]. Most functional tasks in daily life require balance control in the horizontal and vertical directions. Impaired balance is associated with poor motor control, the ability for one to maintain their balance and body orientation during locomotion [11]. Previous studies demonstrated that patients with LBP may have impaired motor control [12–14], which would further affect their balance performance and motor behaviour.

Balance dysfunction in the aging population is based on knowledge of the normal aging processes, loss of sensory elements, and loss of musculoskeletal function [15]. Balance performance declines with age due to biological changes (e.g. mobility, physical inactivity), which in turn could lead to falls [16, 17]. LBP is known to be an independent risk factor for recurrent falls in older women [18].

What is the effect of aging combined with LBP? Here we aimed to conduct a systematic review and meta-analysis to understand the effects of low back pain in elderly people with the ultimate goal of providing better clinical research and treatment guidelines.

## Methods

### Literature search strategy

This review was conducted according to the guidelines outlined in the Preferred Reporting Items for Systematic Reviews and Meta-analysis (PRISMA) statement [19]. Two independent investigators screened the titles and abstracts of the retrieved studies to identify those appropriate for full-text review. Subsequently, they independently assessed the papers in full to identify the studies to be included in the analysis. Any disagreements about inclusion were resolved by discussion and through arbitration by a third reviewer.

### Selection criteria

We included articles that:

1. Participants included elderly people with a mean age of ≥60 years who had chronic LBP;
2. For case-control study, participants included a control group of individuals healthy without LBP;
3. Outcome measures included a measure of balance performance (e.g. balance and gait) that uses highly valid and reliable methods (such as static and dynamic posturographic analyses, centre of pressure [COP] analysis, centre of gravity analysis, and timed up and go test) to access their dynamic or static balance or balance performance. All the articles had to have been available in the English language and published in full within a peer-reviewed journal;
4. studies that scored ≥4 on the Cross-sectional/Prevalence Study Quality scale (Agency for Healthcare Research and Quality, AHRQ) [20];
5. were written in English.

The following exclusion criteria were used:

1. Articles appeared only in abstract format or included insufficient detail to gauge study quality and extract results;
2. The articles were case reports or experimental studies.

### Study selection

**Fig. 1:**
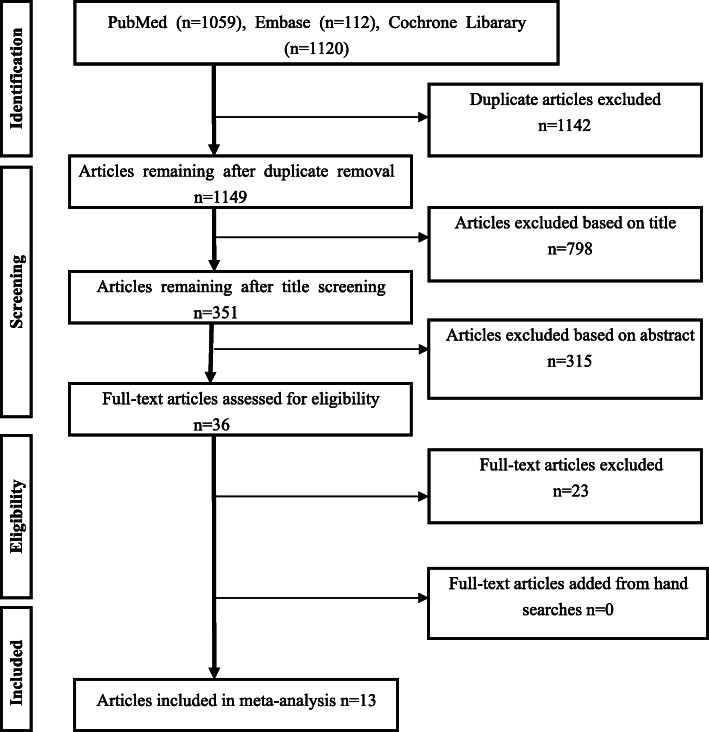
PRISMA flow chart of the article screening and selection process

The search strategy is displayed in Fig. 1. Two reviewers independently screened all abstracts of articles potentially meeting the inclusion criteria. The full texts of those articles were subsequently reviewed. The reviewers then met with the entire review team and resolved any disagreements via consensus. The initial search yielded 2291 publications. Following the title and abstract screening, 35 full-text articles were retrieved. The full-text review was completed to determine final inclusion; 13 articles case-control studies [21–33] met the inclusion criteria.

### Quality assessment

Quality was assessed using the Cross-sectional/Prevalence Study Quality (AHRQ) [20], which has 11 tests and a total score of 11 points. The two researchers independently evaluated all studies that met the inclusion criteria; there were no significant intergroup differences.

### Data extraction

For each study that met the full inclusion and exclusion criteria, information regarding study design and outcome measures (e.g. COP, one-leg stance time) were extracted. The major results of each study focusing on balance function were briefly summarised. The meta-analysis data were collected from the results sections and tables of the manuscripts. The graphs were also used to extrapolate the data. If it was impossible to collect the data from the manuscript, the corresponding author of the manuscript was contacted twice before the study was excluded.

## Results

### Search findings

Figure 1 illustrates the search findings. The initial search yielded 2291 articles. After the two round of screening, 13 case-control studies that compared balance performance for elderly adults with LBP and healthy participants were remained. Data from the included studies are summarized in Table 1. Six studies use the COP parameters to evaluated the the balance performance of the participants ^[23,25.26,27,29,32]^. Two studies use the relative proprioceptive weighting (RPW) [22, 24]. Three studies use the TUG test [21, 26]. And three studies use the gait parameters [28, 30, 31].

**Table 1:** Basic characteristics of included case-control studies

References Design		Basic data of Participant					Balance task	Outcome measure (balance performance)
		LBP		Health		Source of participants		
		Age (mean ± SD)	N	Age (mean ± SD)	N			
Yi-Liang(2015) [21]	case-control	60.5(4.1)	13	59.7 (3.0)	13	local communities and affiliated hospital	Single-leg standing	TUG STS
Ito(2018) [22]	case-control	75.5 (5.1)	28	73.7 (5.7)	46	Department of Physical Medicine and Rehabitation	eyes closed Muscle Vibration	RPW
Brumagne (2004) [23]	case-control	63	10	63	10	Department of Physical Medicine and Rehabilitation	1. control (no vibration); 2. bilateral vibration of thetriceps surae tendons; 3. bilateral vibration of the Tibialis anterior tendons; 4. bilateral vibration of the Paraspinal muscle bellies	COP
Ito(2017) [24]	case-control	76.7 (4.2)	47	73.8 (4.9)	64	NationalCenter for Geriatric and Gerontology	Muscle Vibration	RPW
Lee(2016) [25]	case-control	64.5 (5.7)	30	66.2 (4.5)	26	University Hospital, local communities, and around the campus	Postural perturbation	COP
Iversen(2009) [26]	case-control	75.5 (5.1)	28	73.7 (5.7)	46	tertiary care spine center	static standing	TUG COP
Kendall(2018) [27]	case-control	82.4 (4.6)	24	81.1 (4.3)	19	a preventative home visit program	static standing	COP
Sung(2017) [28]	case-control	65.1 (13.5)	51	63.6 (15)	59	community	walk	gait parameters
Lihavainen(2010) [29]	case-control	80.6 (4.8)	291	80.1 (4.4)	314	all the inhabitants of thecity in Finland	static standing eyes open eyes close Feet together	COP
Champagne(2012) [30]	case-control	68.9 (6.6)	15	69.4 (6.4)	15	local community	–	TUG One-leg stance Walking speed
Hicks(2018) [31]	case-control	69.3 (6.7)	54	71.1 (6.8)	54	community	walk	gait parameters
Silva(2016) [32]	case-control	70.0(8)	10	73.0 (7)	10	local community	one-leg stance	COP
Kato(2019) [33]	case-control	77.4 (4.2)	21	78.1 (4.4)	17	outpatient of hospital	one-leg standing	standing time

*N* number of participants in the study, *COP* centre of pressure, *RPW* relative proprioceptive weighting, *STS* sit-to-stand test, *TUG* timed up and go test. *LBP* low back pain participants, *Health* healthy participants without low back pain

In order to efficiently reduce the risks of bias, the studies had to score ≥ 4 on AHRQ scale to be included in the review. The individual scores attained by the studies using the AHRQ scale are reported in Tables 2. The average AHRQ score for the 13 included studies was computed to be 5.6 out of 11, indicating fair quality of the overall studies.

**Table 2:** Quality assessment of included studies

Agency for Healthcare Research and Quality												
Study	Item											Score
	Define source of information (survey or review)	List inclusion and exclusion criteria for exposed and unexposed subjects (cases and controls) or refer to previous publications	Indicate time period used to identify patients	Indicate whether subjects were consecutive if not population- based	Indicate if evaluators of subjective components of study were masked to, Other aspects of the status of the participants	Describe any assessments undertaken for quality assurance purposes	Explain any patient exclusions from analysis	Describe how confounding variables were assessed or controlled for	If applicable, explain how missing data were handled in the analysis	Summarise patient response rate and completeness of data collection	Clarify what follow-up, if any, was expected and the percentage of patients for whom incomplete data or follow-up was obtained	
Yi-Liang (2015) [20]	Y	Y	Y	U	U	Y	N	Y	N	Y	N	6
Ito (2018) [21]	Y	Y	Y	U	N	Y	N	N	U	Y	N	5
Brumagne (2004) [22]	Y	N	N	N	U	Y	N	Y	U	Y	N	4
Ito (2017) [23]	Y	Y	Y	U	N	Y	N	Y	U	Y	N	6
Lee (2016) [24]	Y	Y	Y	U	N	Y	N	Y	N	Y	N	6
Iversen (2009) [25]	Y	Y	Y	U	U	Y	N	Y	N	Y	N	6
Kendall (2018) [26]	Y	Y	Y	U	N	Y	N	Y	N	Y	N	6
Sung (2017) [27]	Y	Y	Y	Y	N	Y	N	Y	N	Y	N	7
Lihavainen (2010) [28]	Y	Y	N	Y	N	N	Y	N	Y	Y	N	6
Champagne (2012) [29]	Y	Y	N	Y	N	Y	N	N	Y	Y	N	6
Hicks (2018) [30]	Y	Y	Y	U	N	Y	N	N	N	Y	N	5
Silva (2016) [31]	Y	Y	Y	Y	N	Y	N	N	N	Y	N	6
Kato (2019) [32]	Y	Y	N	Y	N	Y	N	U	N	Y	N	5

*N* NO, *Y* YES, *U* UNCLEAR

### Outcomes

#### One-leg stance

A total of four articles used one-leg stance time to assess the balance function of patients with LBP and their healthy counterparts; however, one just calculated the number of people who stood on a single leg for 20 s; therefore, we extracted data from three articles. No significant difference was noted between the two groups (Table 3).

**Table 3:**
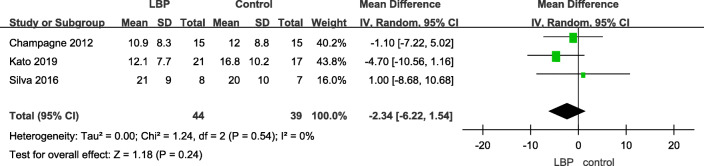
One-leg stance

#### COP area

A total of four studies used COP parameters to measure balance performance, which was recognised as a valid and reliable method. The larger the COP area was, the worse the balance performance was. Older adults with LBP had a longer path length and larger area of COP movements than older adults without LBP (Table 4).

**Table 4:**
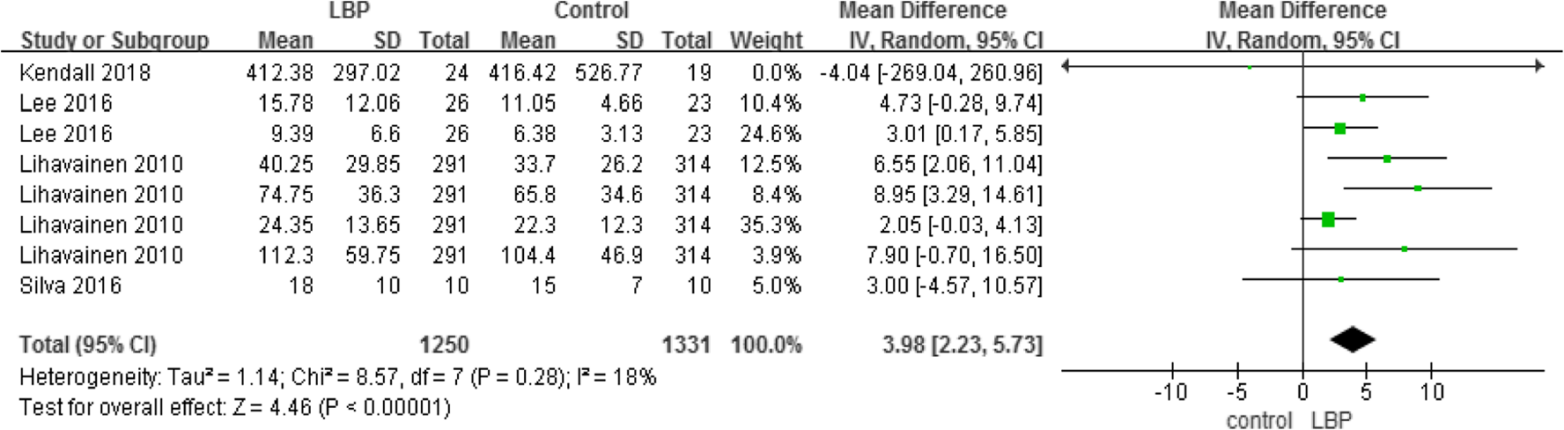
Centre of pressure area

#### COP anteroposterior velocity, mediolateral velocity, and anteroposterior range

A total of four studies used COP sway velocity parameters to measure motor control (Tables 5 and 6), while two studies used COP sway range parameters to measure motor control (Table 7). The higher the COP sway velocity and the longer path length in the anteroposterior direction was, the more unstable  the individual was. The three parameters also demonstrated that older adults with LBP would have higher velocity and larger COP movements than older adults without LBP.

**Table 5:**
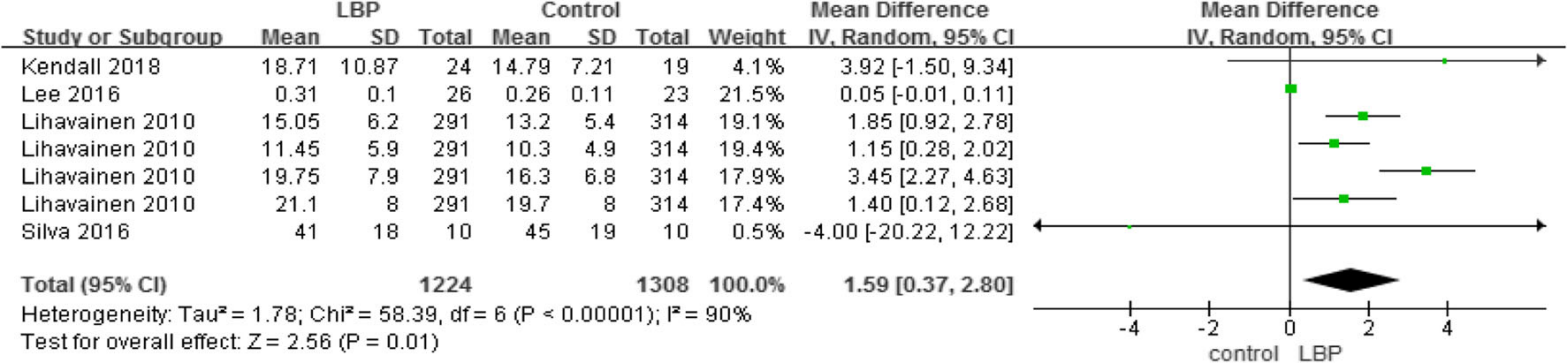
Centre of pressure, anteroposterior velocity

**Table 6:**
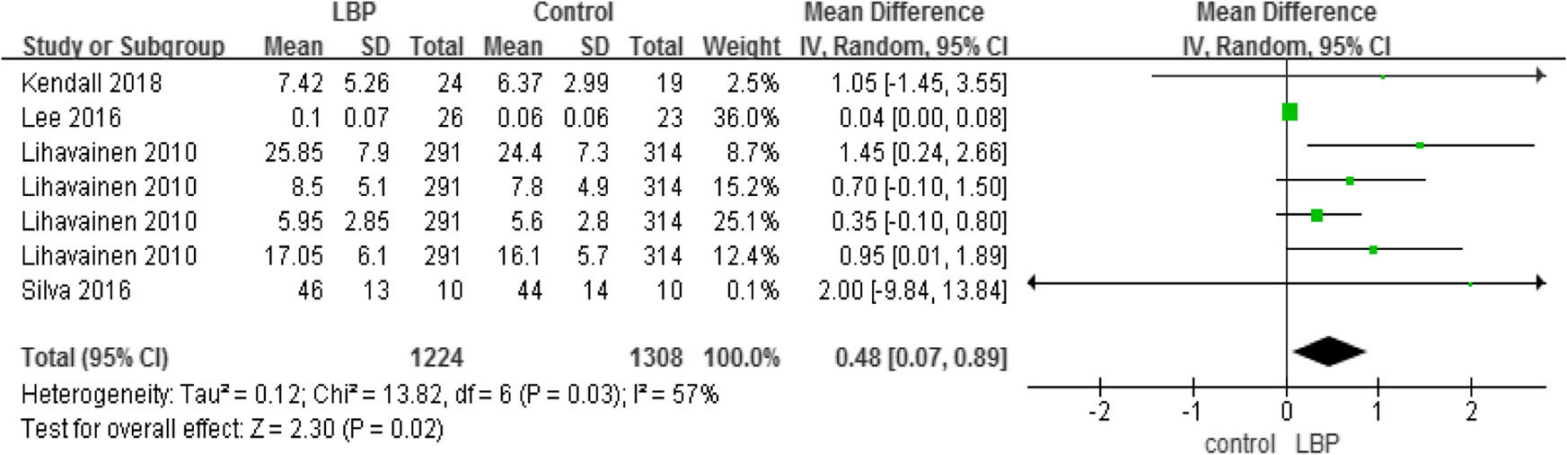
Centre of pressure, mediolateral velocity

**Table 7:**
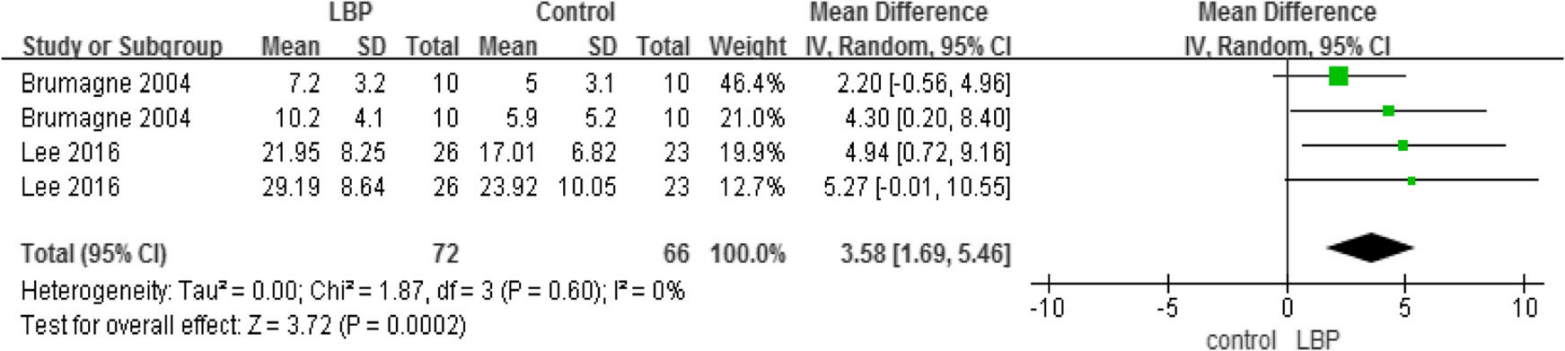
Centre of pressure, anteroposterior range

#### Gait (speed) and TUG

A total of three studies used the gait test (Table 8) and two studies used the TUG (Table 9) to compare the dynamic balance between individuals with LBP and those without LBP. The result showed that, compared to healthy individuals, patients with LBP walked more slowly and needed more time to complete the TUG test.

**Table 8:**
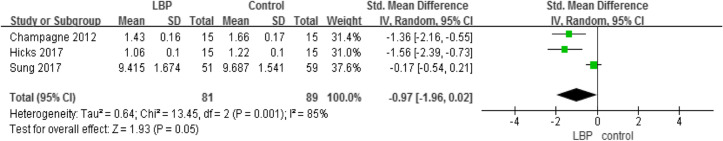
Gait speed

**Table 9:**
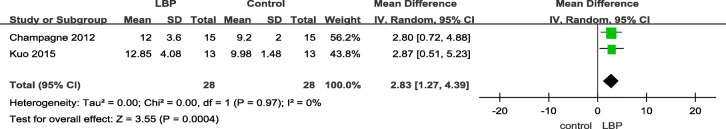
Timed up and go test

#### Relative proprioceptive weighting

Two studies compared the RPW between the two groups but found no significant intergroup difference (Table 10).

**Table 10:**
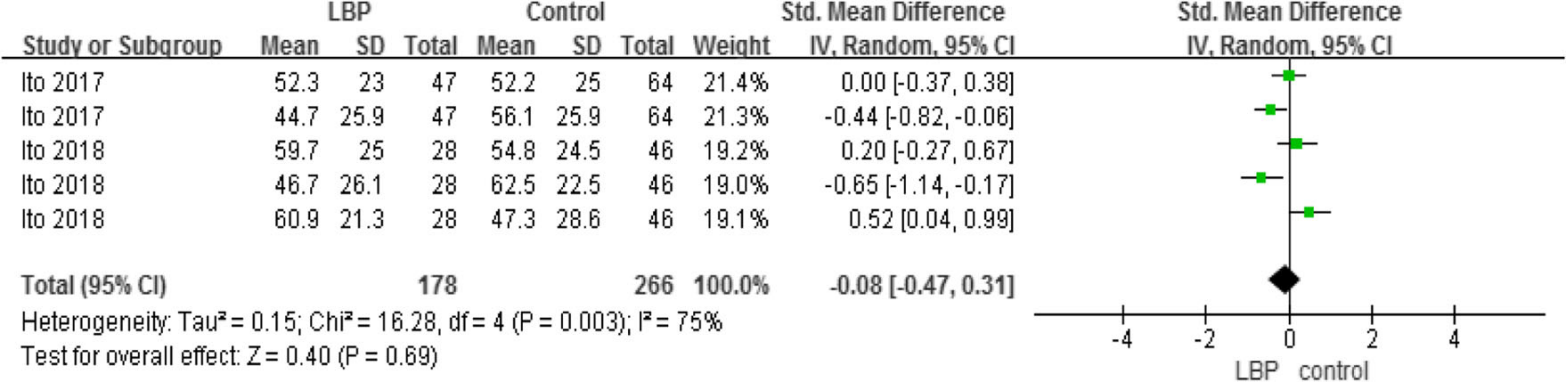
Relative proprioceptive weighting

#### Outcomes measures, risk bias of the studies included in the meta-analysis and sensitivity analysis

Using STATA software to assess the study biases and sensitivity analysis (Table 11), the sensitivity results suggested that our meta-analysis results are relatively stable.

**Table 11:** Outcomes measures, risk bias of the studies included in the meta-analysis and sensitivity analysis

outcomes	References	Design	*P* value	Egger’s test (*P* value)
One-leg stance	Champagne(2012) [30] Silva(2016) [32] Kato(2019) [33]	case-control	*P* = 0.24	0.365
COP area	Lee(2016) [25] Kendall(2018) [27] Lihavainen(2010) [29] Silva(2016) [32]	case-control	*P* < 0.01	0.273
COP AP velocity	Lee(2016) [25] Kendall(2018) [27] Lihavainen(2010) [29] Silva(2016) [32]	case-control	*P* = 0.01	0.929
COP ML velocity	Lee(2016) [25] Kendall(2018) [27] Lihavainen(2010) [29] Silva(2016) [32]	case-control	*P* = 0.02	0.161
COP AP range	Brumagne(2004) [23] Lee(2016) [25]	case-control	*P* < 0.01	0.184
Gait	Sung(2017) [28] Hicks(2018) [31] Champagne(2012) [30]	case-control	*P* = 0.05	0.037
TUG	Yi-Liang(2015) [21] Iversen(2009) [26] Champagne(2012) [30]	case-control	*P* < 0.01	0.317
RPW	Ito(2018) [22] Ito(2017) [24]	case-control	*P* = 0.69	0.682

*COP* centre of pressure, *RPW* relative proprioceptive weighting, *AP* anteroposterior, *ML* mediolateral, *TUG* timed up and go test

## Discussion

This meta-analysis, which identified 13 case-control studies that compared balance performance for elderly adults with LBP and healthy participants. The qualities of all the case-control studies were moderate. The risk of bias was assessed for each article using the Cochrane Collaboration recommendations, the sensitivity results suggested that our meta-analysis results seem to be stable.

To our knowledge, this is the first systematic review and meta-analysis to focus on LBP and balance performance in elderly people. This systematic review aimed to estimate the effect of LBP on balance performance in elderly people. Our results demonstrated that elderly people with LBP have poorer balance performance than those without LBP. With the rapidly aging society, the proportion of elderly patients with chronic LBP is increasing annually, which lead to bad moods, functional inactivity, a decrease in quality of life, and an increase in fall risk. [34]. It is necessary to provide effective intervention measures to improve elderly peoples’ quality of life and reduce the economic losses and physical and emotional trauma caused by chronic LBP.

The aging process results in changes in the central nervous system, peripheral nervous system, and the musculoskeletal system [35, 36]. Pain itself has a wide range of effects on motor function [37]. People who experience chronic pain display changes in motor patterns, exercise coordination, and the ability to maintain stability in response to external disturbances. Pain induces spinal motility restrictions, lumbar proprioceptive losses, weakening of lower-extremity sensory feedback, and trunk muscle weakness and atrophy [26, 28, 29, 32]. Thus, when aging is combined with LBP, balance performance becomes worse. However, almost 90% of articles to date focused on young and middle-aged people. It is conceivable that conditions associated with younger and middle-aged people are more optimistic regardless of the balance performance or responsive to treatment. It is also possible that older adults with LBP should be subjected to different assessments and interventions than younger adults to account for the differences in therapeutic approaches and treatment outcomes. Given the age disparities in LBP people, in addition to solving the pain issue, it is important to focus on the balance performance of older individuals. As we all know, balance control with age is among the major risk factors for falls, which is a difficult problem that the world faces, especially as the population continues to age [38].

Poor balance performance in elderly people with LBP means they could not perform accurate movements and ambulation [39, 40], which in turn affected their physical activities. In the present study, TUG, one-leg stance, postural sway, and gait are reliable and valid fall-risk assessments [41–44], poor outcomes on the TUG and postural sway tests may indicate an increased risk of falling that could lead to disastrous consequences**.** Problems with balance performance were also reportedly associated with fall risk [45]. Physical therapists in the clinical setting should be aware of an increased risk of falling for their patients with LBP.

There is some evidence that LBP impacts the equilibrium of older individuals. However, only one study in this review assessed reactive balance control, which assessed the postural responses to a suddenly released pulling force in older adults with LBP [24]. The results showed older adults with LBP had poorer postural responses in delayed reaction, larger displacement, higher velocity, longer path length, and greater COP sway area compared to the older healthy controls. The outcome parameters assessed in Lee et al’s [24] study were similar to those used in present study. Sudden postural perturbations are very common during everyday life, such as pulling an object that might suddenly move or open, poorer reactive balance control is important to maintain balance in the sudden postural perturbation, which could reduce the falling risk. Besides the reactive balance control, it is also very common for postural tasks to be accompanied with cognitive tasks (e.g., making a telephone call while walking) in daily life. Understanding of the effects of dual tasks on static and dynamic balance performance among older individuals with LBP could help reduce the occurrence of falls for the elderly people. However, none study assessed the effect of dual tasks on balance performance in older individuals with LBP. Future studies must focus more on this issue, especially on motor performance or balance function in these patients. The results of this review depended on the outcome measures examined in the reviewed articles, and the small sample sizes may have limited the power of our findings. Therefore, future research should include adequate sample sizes and must be combined with myoelectric and neural electrical activity and gait analyses to evaluate dynamic-static equilibrium and reactive balance, which could better reflect the effect of the central nervous system on peripheral control.

### Study limitations

This review included only case-control studies. Furthermore, the present review did not incorporate non-English studies. This may limit the validity of our findings and must be taken into consideration when interpreting its overall generalizability. The limited number of case-control studies did not allow a subgroup meta-analysis. However, we achieved the main aim of our review, which was to estimate the effect of LBP on balance performance in elderly people.

## Conclusion

In summary, the study results indicate evidence in favour of a negative effect of LBP on balance performance in elderly people with LBP. Future studies should focus on the mechanisms and effective interventions for abnormal balance control in elderly people with LBP.

## Data Availability

Not applicable.

## Abbreviations

LBP: Low back pain
COP: Centre of pressure
RPW: Relative proprioceptive weighting
AP: Anteroposterior
ML: Mediolateral
TUG: Timed up and go test
STS: Sit-to-stand test
AHRQ: Cross-sectional/Prevalence Study Quality
PRISMA: Systematic Reviews and Meta-analysis
